# A multiple shoot induction system for peptide-mediated gene delivery into plastids in *Arabidopsis thaliana*, *Nicotiana benthamiana*, and *Fragaria*×*ananassa*

**DOI:** 10.5511/plantbiotechnology.23.0501a

**Published:** 2023-12-25

**Authors:** Masaki Odahara, Most Tanziman Ara, Remi Nakagawa, Yoko Horii, Shougo Ishio, Shinjiro Ogita, Keiji Numata

**Affiliations:** 1 Biomacromolecule Research Team, RIKEN Center for Sustainable Resource Science, Wako, Saitama 351-0198, Japan; 2 Resources Group, Tsukuba Research Institute, Sumitomo Forestry Co., Ltd., Tsukuba, Ibaraki 300-2646, Japan; 3 Department of Local Resources, Faculty of Bioresource Sciences, Prefectural University of Hiroshima, Shobara, Hiroshima 727-0023, Japan; 4 Department of Material Chemistry, Kyoto University, Kyoto, Kyoto 615-8510, Japan

**Keywords:** liquid culture, multiple shoot induction, peptide-mediated gene delivery, plastid transformation

## Abstract

The plastid is a promising target for the production of valuable biomolecules via genetic engineering. We recently developed a plastid-specific gene delivery system for leaves or seedlings using KH-AtOEP34, a functional peptide composed of the polycationic DNA-binding peptide KH and the *Arabidopsis thaliana* plastid-targeting peptide OEP34. Here, we established a liquid culture system for inducing multiple shoots in the model plants *A. thaliana* and *Nicotiana benthamiana* and the crop plant strawberry (*Fragaria*×*ananassa*) and tested the use of these plant materials for peptide-mediated gene delivery to plastids. Our liquid culture system efficiently induced multiple shoots that were enriched in meristems. Using these meristems, we performed KH-AtOEP34-mediated gene delivery to plastids and tested the delivery and integration of a cassette composed of the spectinomycin resistance gene *aadA*, the *GFP* reporter gene, and sequences homologous to plastid DNA. Genotyping PCR revealed the integration of the cassette DNA into plastid DNA several days after delivery in all three plants. Confocal laser scanning microscopy and immunoblotting confirmed the presence of plasmid-derived GFP in the plastids of meristems, indicating that the plasmid DNA was successfully integrated into plastid DNA and that the cassette was expressed. These results suggest the meristems developed in our liquid culture system are applicable to peptide-mediated delivery of exogeneous DNA into plastids. The multiple shoots generated in our liquid novel culture system represent promising materials for in planta peptide-mediated plastid transformation in combination with spectinomycin selection.

## Introduction

Plastid transformation has several advantages over nuclear transformation, as it is effective for high-level protein production and site-specific integration of transgenes without inducing post-transcriptional gene silencing. While genome editing in plastids was recently achieved using plastid-target base editors fused with TALE (transcription activator-like effector) units (Kang et al. 2021; Nakazato et al. 2021), conventional genetic transformation via gene targeting mediated by homologous recombination is still the major method for plastid transformation. The first successful plastid transformation was performed in the green unicellular alga Chlamydomonas (*Chlamydomonas reinhardtii*) (Boynton et al. 1988). This technique was subsequently expanded to model plants including tobacco (*Nicotiana tabacum*) (Svab et al. 1990) and Arabidopsis (*Arabidopsis thaliana*) (Ruf et al. 2019; Sikdar et al. 1998; Yu et al. 2017), as well as crop plants including potato (*Solanum tuberosum*) (Sidorov et al. 1999) and tomato (*S. lycopersicum*) (Ruf et al. 2001).

Biolistic transformation, the principle method for plastid transformation via homologous recombination, involves the bombardment of submicrometer-sized metal particles coated with DNA into plant materials to deliver the DNA into cells. However, this well-established plastid transformation method requires the use of special equipment and high-pressure gas, and can cause chromosomal instability (Liu et al. 2019). Polyethylene glycol (PEG)-mediated transformation, which introduces DNA into protoplasts via by the action of PEG, is another method used for stable plastid transformation (O’Neill et al. 1993). However, few plants are capable of regeneration from protoplasts into intact plants. With both methods, the delivered DNA is not specifically targeted to the plastids. Instead, plastid transformants are selected based on the presence of a selectable marker gene such as the aminoglycoside-3″-adenylyltransferase gene *aadA*, which confers resistance to spectinomycin/streptomycin.

Recent advances in the development of nanomaterials have facilitated selective gene delivery into plastids. Functional peptides, which are short peptides ∼50 amino acids in length with specific functions, have been used as carriers for the delivery of biomacromolecules into plant cells and plastids in the cells. Such functional peptides include CPPs, which can pass through the cell membrane; polycationic peptides, which bind to negatively charged biomolecules such as nucleic acids; and organelle-targeting peptides, which target biomolecules to plastids and/or mitochondria (Watanabe et al. 2021). These functional peptides are tandemly arranged as fusion peptides for the efficient delivery of biomolecules into plant cells (Lakshmanan et al. 2013).

The fusion peptide KH-AtOEP34 is composed of the polycationic peptide KH9 and the plastid-targeting peptide from chloroplast-localized OUTER ENVELOPE PROTEIN 34 (OEP34) from Arabidopsis (Yoshizumi et al. 2018). KH-AtOEP34 has been used to deliver pDNA to various types of plastids including chromoplasts, amyloplasts, and chloroplasts (Thagun et al. 2019). The pDNA is transiently integrated into the DNA of the target plastid via homologous recombination (Yoshizumi et al. 2018). Peptide-based micelles containing a chloroplast-targeting peptide have also been used for plastid-specific DNA delivery in combination with a cell wall-relaxation reagent (Miyamoto et al. 2022). Stable plastid transformants have been obtained by combining KH-AtOEP34-mediated DNA delivery and regeneration under the appropriate selection conditions in tobacco, rice (*Oryza sativa*), and kenaf (*Hibiscus cannabinus*) (Odahara et al. 2022). Single-walled carbon nanotubes (SCNTs) are also used as carriers for the delivery of biomacromolecules into plant organelles. Chitosan-complexed SCNTs have been utilized for plastid-selective gene delivery in a variety of plants (Kwak et al. 2019), and SCNTs modified with functional peptides have been used for efficient gene delivery into plant mitochondria (Law et al. 2022). Notably, these nanomaterial-based gene delivery methods do not require any biolistic equipment or chemical treatments, making them suitable for quick and easy gene delivery into plant cells and plant organelles.

In the present study, we generated multiple shoots from the model plants Arabidopsis and *Nicotiana benthamiana* and the crop plant strawberry (*Fragaria*×*ananassa*) via a newly developed liquid culture system and tested their suitability as materials for fusion peptide-mediated plastid transformation. To date, shoots induced from calli have been used for genetic transformation of Arabidopsis (Sikdar et al. 1998), *N. benthamiana* (Davarpanah et al. 2009), and *F.*×*ananassa* (Amjad and Srivastava 2006), including their plastid transformation. Multiple shoots produced by our technique can be used to generate intact plants without the need for tissue culture or regeneration, making them suitable for simple, rapid plastid transformation in planta. Moreover, the use of liquid medium sometimes leads to higher rates of multiplication and improved growth compared to semi-solid medium (Ara et al. 2020; Ogita et al. 2008). In addition, since plant materials are submerged in peptide solution during fusion peptide-mediated plastid transformation (Watanabe et al. 2021), we reasoned that the multiple shoots produced by our liquid culture system should be compatible with the fusion peptide method. Indeed, our results indicate that multiple shoots produced by our liquid culture method are promising materials for fusion peptide-mediated plastid transformation in both crops and model species.

## Materials and methods

### Explant preparation and multiple shoot induction in liquid medium

Arabidopsis (*Arabidopsis thaliana*, accession Columbia-0 [Col-0]), *Nicotiana benthamiana*, and *Fragaria*×*ananassa* cv. Benihoppe were used in this study.

Arabidopsis seeds were surface sterilized with 70% (v/v) ethanol for 3 min and then with 20% (w/v) sodium hypochlorite for 20 min, followed by rinsing with sterilized distilled water. The seeds were placed on full-strength Murashige and Skoog (MS) medium (Sigma-Aldrich Co., USA) containing 3% (w/v) sucrose without growth regulators in a 90-mm diameter disposable Petri dish. The pH was adjusted to 5.8 using KOH, and 0.25% (w/v) Phytagel was added to the medium as a solidifying agent before autoclaving. Ten-day-old seedlings were transferred to 10 ml of liquid Gamborg’s B5 medium (Nihon Pharmaceutical Co., Ltd., Japan) supplemented with 3% (w/v) sorbitol and 2.0 mg l^−1^ BAP (shoot induction medium [SIM]) in an 18-mm glass test tube to induce multiple shoot formation. The glass tubes were placed on a disc-type rotator (1 rpm). The shoots were then multiplied in 20 ml of liquid Gamborg’s B5 medium supplemented with 3% (w/v) trehalose and 0.2 to 2.0 mg l^−1^ BAP (shoot multiplication medium [SMM]) in a 25-mm glass test tube. The multiple shoots were subcultured once per week. Arabidopsis cultures were maintained in the same growth chamber at 22°C.

*N. benthamiana* seeds were surface sterilized with 70% (v/v) ethanol for 1 min and then with 20% (w/v) sodium hypochlorite for 20 min, followed by rinsing with sterilized distilled water. The seeds were placed on half-strength MS medium containing 3% (w/v) sucrose without growth regulators in a 90-mm diameter disposable Petri dish. The pH was adjusted to 5.8, and 0.25% (w/v) Phytagel was added in the medium as a solidifying agent before autoclaving. Apical buds were excised from 2-week-old seedlings and cultured in multiple shoot induction medium (SIM; 10 ml of liquid half-strength MS medium supplemented with 0.1 to 0.2 mg l^−1^ BAP) in a 25-mm glass tube. One-week-old elongated shoots were transferred to SIM (50 ml of liquid medium in a 300-ml conical flask). The multiplied shoots were subcultured every two weeks.

For *F.*×*ananassa*, runner tips were collected, surface sterilized with 70% (v/v) ethanol for 30 min and then with 1% (w/v) sodium hypochlorite for 10 min, and rinsed with sterilized distilled water. The meristems were cultured on 25 ml of half-strength MS medium supplemented with 3% (w/v) sucrose and 0.05 mg l^−1^ BAP, pH 5.8, solidified with 0.8% (w/v) agar in a 90-mm diameter disposable Petri dish. Seventy-day-old regenerated microshoots were transferred into a 300-ml conical flask containing 30 ml of liquid MS medium supplemented with 2 mg l^−1^ BAP, 0.5 mg l^−1^ kinetin (KIN; Sigma-Aldrich Co., USA), and/or 0.25 mg l^−1^ GA_3_ (Sigma-Aldrich Co., USA) for shoot multiplication.

All in vitro cultures of *F*.×*ananassa* and *N. benthamiana* were maintained at 25°C under long-day conditions (16-h light/8-h dark photoperiod) in an LH-410S growth chamber (NK System, Japan). For *Arabidopsis*, cultures were maintained at 22°C under long-day condition in an LH-410S (NK System, Japan).

### Isolation of shoot apical meristems

Shoot apical meristems (SAMs) were carefully isolated from the shoot tips of each plant using sharp forceps and a blade. The meristems were dipped into shoot culture medium in a Petri dish to maintain proper moisture and nutrient conditions. The meristem isolation procedure was performed on the same day as transformation. Each isolated meristem was maintained in a well of a 6-well microplate containing 2 ml liquid culture medium. Filter paper was placed at the base of the well by folding it in the middle to prevent the meristem from fully sinking into the medium.

### Plasmid DNA construction

To target plastid DNA, a pDNA construct was generated for each plant species. For Arabidopsis, the left and right homology arms targeting the *trnV*-*rps12*/*7* region were amplified with p1 (5′-CGGTACCCGGGGATCAAAGGAGGTGATCCAGCC-3′) and p2 (5′-GAAGCTTATCGGATCGATCTTTGGCGCAAGAATAA-3′) and p3 (5′-GATCTTCCTATTTCCAAAGG-3′) and p4 (5′-CGACTCTAGAGGATCGATCTCCCTCCAAACCGT-3′) from Arabidopsis Col-0 genomic DNA, respectively. The *aadA* and *GFP* cassettes, P*rrn*-*aadA-*T*psbA* and P*psbA*-*GFP-*T*rps16* (regulatory sequences are derived from *N. tabacum* chloroplast DNA) (Odahara et al. 2022), amplified with p5 (5′-GATCCGATAAGCTTCGAATA-3′) and p6 (5′-GGAAATAGGAAGATCGATCTGACAATTGGTGGCG-3′) were inserted between the homology arms using an In-Fusion HD Cloning Kit (Takara, Japan) to obtain pPTTF_At. For *N. benthamiana*, a typical construct comprising homology arms that target an intergenic region between *trnV* and *rps12*/*7* of *N. tabacum* plastid DNA (Zoubenko et al. 1994) and the *aadA* and *GFP* cassettes between the homology arms was used to obtain pPTTF_Nb. For *F.*×*ananassa*, a plastid DNA sequence from *F.*×*ananassa* genomic DNA was amplified with p7 (5′-CGGTACCCGGGGATCGGGAACGAATTCACCGCC-3′) and p8 (5′-CGACTCTAGAGGATCGTAAGGCAGAGTTTGGTTT-3′) and subcloned into pUC19 using an In-Fusion HD Cloning Kit. The *aadA* and *GFP* expression cassettes amplified with p9 (5′-CTTGCGCCAAAGATCCGATAAGCTTCGAATATAGCTCTTC-3′) and p10 (5′-GGAAATGATAAGATCTGACAATTGGTGGCGGCC-3′) were then inserted into the BglII site of the plastid DNA sequence to target the *trnV*-*rps12*/*7* region of *F.*×*ananassa* plastid DNA to obtain pPTTF_Fa.

### Measurement of peptide-DNA complexes

The peptides KH-AtOEP34 and BP100 (Supplementary Table S1) were synthesized according to Thagun et al. (2019). Plasmid DNA+KH-AtOEP34 (pDNA+CTP) complexes were formed at different N/P ratios (the molar ratio of amine groups [NH3^+^] in the peptides to that of negatively charged groups [PO_4_^−^] in the pDNA) by adding different amounts of KH-AtOEP34 peptide to 10 µg of pDNA in water to a final volume of 100 µl. The solutions were mixed by pipetting and incubated at 25°C for 30 min without shaking. After incubation, different amounts of the CPP BP100 were added to the pDNA+CTP complex solution to form pDNA+CTP+CPP complexes at different N/P ratios. The solutions were mixed by pipetting and incubated at 25°C for 30 min without shaking. The pDNA+KH-AtOEP34 or pDNA+KH-AtOEP34/BP100 complex solutions were then diluted in 700 µl of water. The particle size, polydispersity index (PDI), and surface charge of the complexes were determined using a Zetasizer Nano (Malvern Instruments, UK).

### Peptide-mediated delivery of pDNA into plastids

To prepare 100 µl of plasmid-peptide complex for Arabidopsis, 3 µg of pPTTF_At and 2 µg KH-AtOEP34 were mixed to prepare 100 µl of solution at an N/P ratio of 1.0 and incubated at room temperature for 30 min. For *N. benthamiana*, 3 µg pPTTF_Nb and 1 µg KH-AtOEP34 were mixed to prepare 100 µl of solution at an N/P ratio of 0.5 and incubated at room temperature for 30 min. *F.*×*ananassa*, 3 µg pPTTF_Fa and 2 µg KH-AtOEP34 were mixed by pipetting to ensure an N/P ratio of 1.0. After incubation at room temperature for 30 min, BP100 was added to the solution at an N/P ratio of 1.5. Meristems were submerged in the DNA-peptide complex solution and subjected to mechanical vacuum (−0.08 MPa, 1 min) and compression (+0.08 MPa, 1 min) treatment as described in Watanabe et al. (2021). The meristems were maintained under the same culture conditions.

### GFP imaging by confocal laser scanning microscopy

GFP fluorescence in the plastids of *F.*×*ananassa* meristems was observed under an LSM880 confocal laser-scanning microscope (Carl Zeiss, Germany) with chlorophyll autofluorescence. Longitudinal sections of the inside or outside of the meristem and regenerated leaf segments were observed by confocal laser-scanning microscopy (CLSM). The leaf sections were washed twice with water and de-aerated in water prior to observation.

### DNA extraction and genotyping PCR

Genomic DNA for genotyping PCR was extracted from ∼100 mg of meristems collected from each plant using a DNeasy Plant Mini Kit (Qiagen, Germany). Genotyping PCR was performed with the genomic DNA, primers shown in Supplementary Table S2, and PrimeSTAR GXL DNA polymerase (TaKaRa, Japan).

### DNA gel blotting

Genomic DNA digested with AflII was separated by agarose gel electrophoresis and then transferred to a nylon membrane. The blot was hybridized with a probe prepared with a PCR product amplified with p11 (5′-AAGGGGCATGATGACTTGAC-3′) and p12 (5′-CAAGCGTTATCCGGAATGAT-3′) using the AlkPhos Direct Labeling System (Cytiva, USA) and imaged by an image analyzer FUSION SOLO S (Vilber Bio Imaging, France).

### Immunoblot analysis of GFP protein

Meristems frozen in liquid N_2_ were powdered by mortar and pestle, and then suspended in Renilla Luciferase Assay Lysis Buffer (Promega, USA). After centrifugation at 12,000 rpm for 30 min at 4°C, supernatant was subjected for purification using anti-GFP antibody-conjugated magnetic beads according to Thagun et al. (2019). Western blotting was also performed according to Thagun et al. (2019) with 1 : 1,000 rabbit anti-GFP antibody (NB600-308; Novus Biologicals, USA) as the first antibody and 1 : 20,000 horse radish peroxidase conjucated goat anti-rabbit IgG polyclonal antibody (ab6721; Abcam, UK). Using a chemiluminescent substrate (Thermo Scientific, USA), signal was detected by FUSION SOLO (Vilber Bio Imaging, France).

## Results and discussion

### Establishment of a liquid culture system for multiple shoot induction

**Figure 1:**
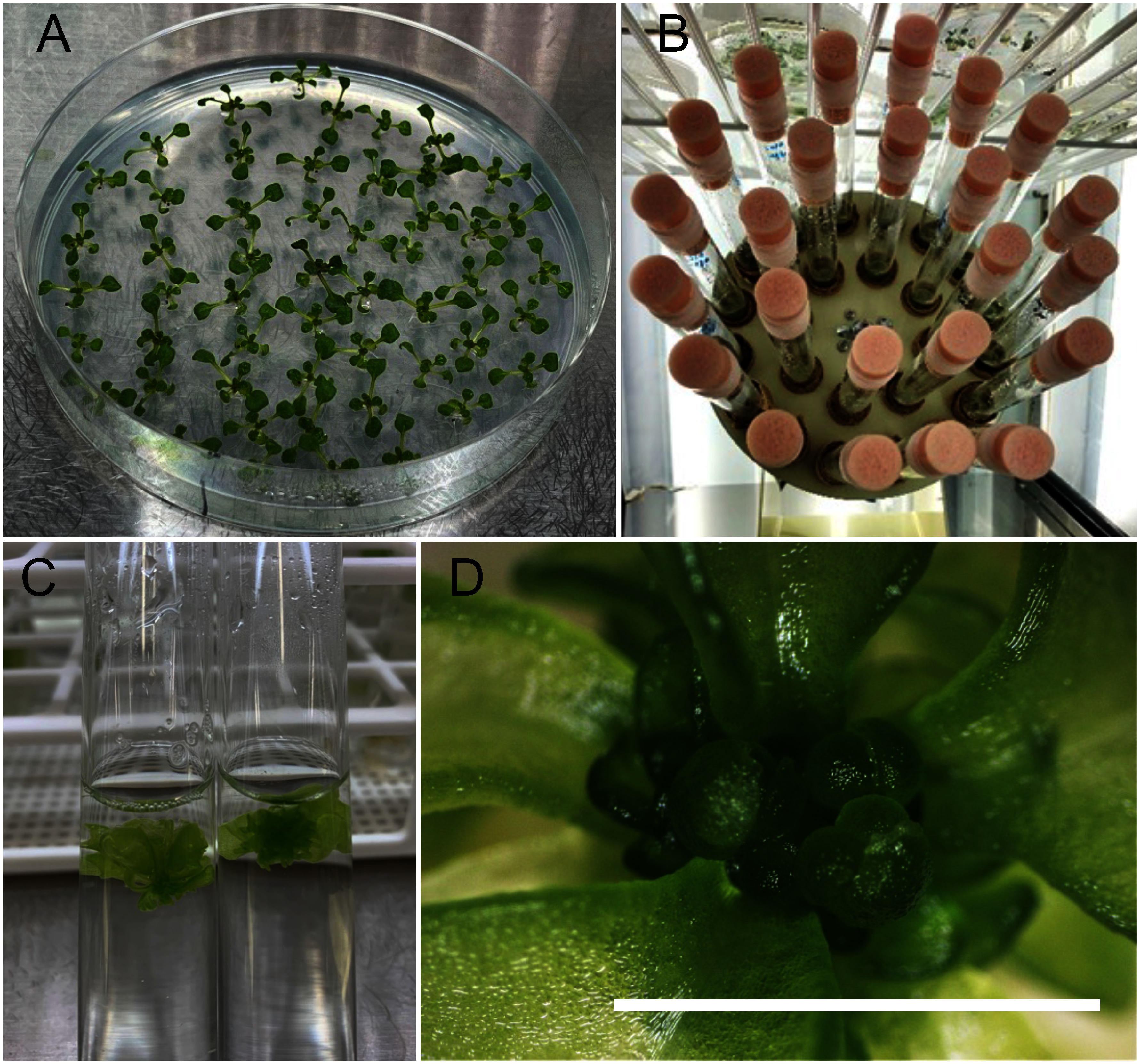
Figure 1. Induction of multiple shoots in Arabidopsis. (A) Ten-day-old seedlings. (B) Induction of multiple shoots in SIM medium using a rotator. (C) Multiple shoots produced after 10 days of cultivation. (D) Observation of meristems under a stereomicroscope. Scale bar, 5 mm.

To establish a rapid and efficient method for plastid transformation of the SAMs of Arabidopsis, *N. benthamiana*, and *F.*×*ananassa*, we established a method for primary culture and multiple shoot induction in liquid culture. For Arabidopsis, we induced multiple shoot formation in liquid Gamborg’s B5 medium containing 3% (w/v) sorbitol with 2.0 mg BAP (Figure 1B). Shoots were subsequently multiplied in liquid Gamborg’s B5 medium containing various concentration of BAP with 3% (w/v) trehalose (Figure 1C, D). As shown in Table 1, 0.2 mg l^−1^ BAP showed the highest efficiency for induction of multiple shoots in Arabidopsis. On the other hand, the addition of maltose instead of sorbitol or trehalose failed to induce multiple shoots despite addition of various concentration of BAP (Supplementary Figure S1), suggesting that the type of sugar is a critical factor for this process in Arabidopsis, as described for the tissue culture of other plants (Liu et al. 2006; Yamaguchi et al. 2011).

**Table 1:** Table 1. Multiple shoot induction of *Arabidopsis*.

BAP (mg l^−1^)	0	0.2	0.5	1	2
Number of shoots (mean±SD)	1.3±0.6	13.3±3.8	8.0±1.0	4.7±0.6	3.3±1.6

**Figure 2:**
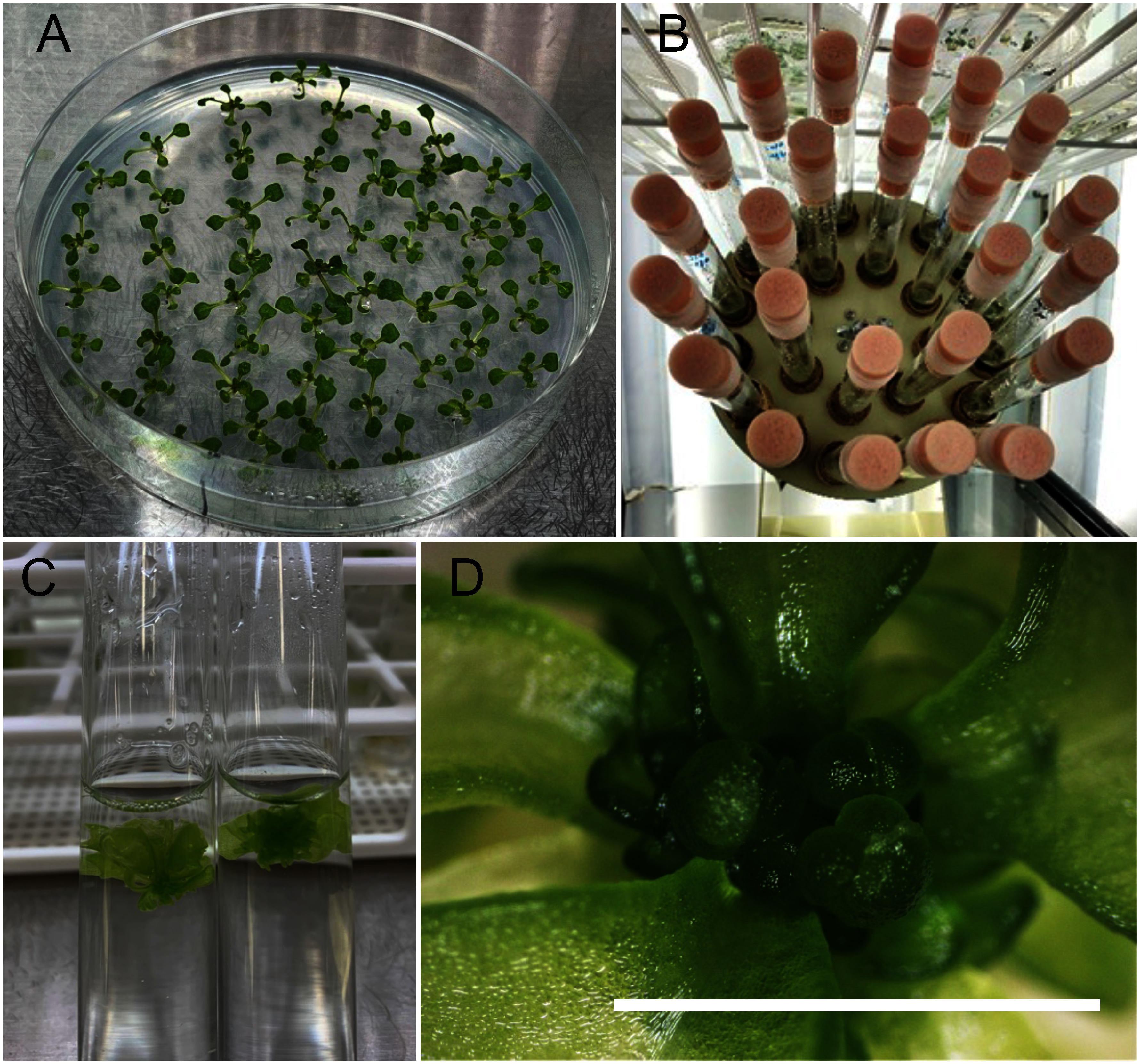
Figure 2. Induction of multiple shoots in *N. benthamiana*. (A) Two-week-old seedlings in a Petri plate containing half-strength MS medium. (B) Shoot elongation after 1 week in SIM. (C) Shoot multiplication after 2 weeks of culture. (D) Isolated shoot apical meristem of *N. benthamiana*. Scale bar, 1 mm. (E) Multiple shoots cultivated for 4 weeks in SIM.

For *N. benthamiana*, we tested multiple shoot induction in medium based on the half-strength MS liquid medium with 3% sucrose, which is normally used for cultivation of *N. benthamiana*. Among the various concentration of BAP tested, 0.1 or 0.2 mg l^−1^ BAP efficiently induced multiple shoot formation in *N. benthamiana* (Table 2 and Supplementary Figure S2), and the shoots were subsequently multiplied in the same medium (Figure 2). This approach offered a more simplified shoot multiplication method for *N. benthamiana* compared to that on solid medium, which requires BAP and 1-naphthaleneacetic acid (NAA) (Carvalho et al. 2008).

**Table 2:** Table 2. Multiple shoot induction of *N. benthamiana*.

BAP (mg l^−1^)	0	0.1	0.2	0.5	1
Number of shoots (mean±SD)	0	5.0±1.0	4.0±0.6	0	0

**Figure 3:**
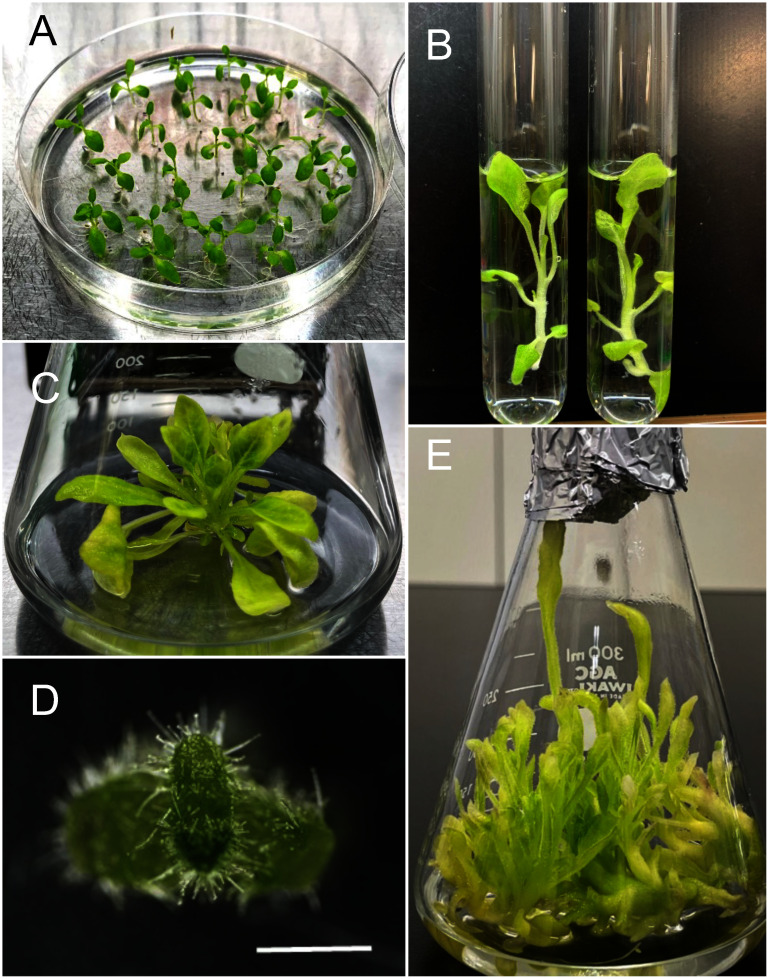
Figure 3. Induction of multiple shoots in *F.*×*ananassa* cv. Benihoppe. (A) Runner tips from soil-grown plants. (B) Developed micro-shoot after 7 weeks of culture on solid medium in a culture box. (C) Typical in vitro multiple shoot culture stock in a conical flask containing liquid MS medium. (D) Isolated shoot apical meristem. Scale bars, 10 mm in (A) and (B), 1 mm in (D).

For *F.*×*ananassa*, multiple shoot induction was achieved in liquid MS medium supplemented with 2 mg l^−1^ BAP, 0.5 mg l^−1^ KIN, and 0.25 mg l^−1^ GA_3_ based on a previously described solid medium used for multiple shoot induction in this plant (Tanziman et al. 2013) (Figure 3). To maximize the number of usable meristems, the *F.*×*ananassa* cultures were transferred to the same medium without GA_3_. The medium without GA_3_ produced fewer multiple shoots compared to medium with GA_3_, while the number of usable meristems was almost the same regardless of GA_3_ addition (Table 3). This result suggested that adding GA_3_ to the medium increases the number of meristematic structures, but most of these structures failed to produce well-developed meristems with leaf primordia. In summary, we established a versatile liquid culture system for multiple shoot induction in Arabidopsis, *N. benthamiana*, and *F.*×*ananassa*, generating multiple shoots that are suitable for peptide-mediated gene delivery.

**Table 3:** Table 3. Number of usable *F.*×*ananassa* meristems suitable for transformation after 4 weeks of cultivation in liquid medium.

Culture medium condition (MS medium)	Rate of multiplication (%)	Number of meristems/explant (mean±SD)	Number of usable meristems/explant (mean±SD)	Remarks
MS	0	1.27±0.44	1.27±0.44	No multiplication
Half-strength MS+0.05 mg l^−1^ BAP	0	1.33±0.47	1.33±0.47	No multiplication
2 mg l^−1^ BAP+0.5 mg l^−1^ KIN	100	15.12±3.14	11.13±2.09	Developed meristem and leaf primordium structure
2 mg l^−1^ BAP+0.5 mg l^−1^ KIN+2.5 mg l^−1^ GA_3_	100	56.6±8.22	13.13±3.66	Immature meristem and leaf primordium structure

Data were collected after 4 weeks of cultivation in liquid medium. Mature meristems were considered to be usable meristems.

### Delivery and integration of pDNA into plastid DNA

**Figure 4:**
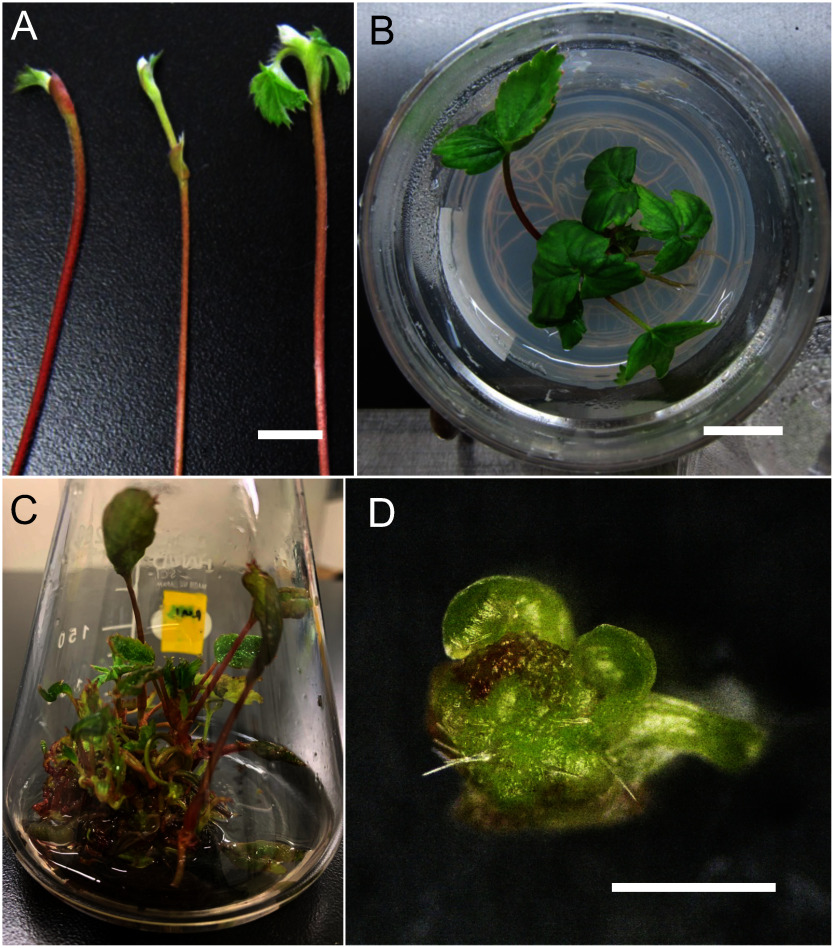
Figure 4. Genotyping PCR of Arabidopsis, *N. benthamiana*, and *F.*×*ananassa* plastids. A. Targeting of plastid DNA by pDNA. The pDNA is integrated into plastid DNA via homologous recombination between the homology arms (left homology arm, LHA; right homology arm, RHA). The primers used in panel B–D are indicated by arrowheads. B–D. Genotyping PCR of peptide-DNA-treated Arabidopsis (B), *N. benthamiana* (C), and *F.*×*ananassa* (D). The left and right border regions were amplified with primers L-Fw/Rv and R-Fw/Rv, respectively. Target DNA products are indicated by arrowheads.

To test the suitability of the multiple shoots generated in liquid culture for peptide-mediated delivery and integration of pDNA into plastid DNA, we used the fusion peptide KH-AtOEP34. The plasmids used to target plastids were composed of sequences homologous to the plastid DNA of each plant species, the *aadA* gene cassette conferring spectinomycin/streptomycin resistance, and the reporter gene *GFP* (Figure 4A). The homologous sequences were designed to target a locus between the 16S rDNA genes *trnV* and *rps12*/*7*, a site that was previously used to integrate foreign DNA into plastid DNA (Daniell et al. 2016; Maliga and Tungsuchat-Huang 2014). Since the *N. benthamiana* plastid DNA sequence was not available in a public database, we used the plastid DNA sequence of the related species *N. tabacum* (Shinozaki et al. 1986) to design the homologous sequence for *N. benthamiana* plastid DNA. We complexed each pDNA with KH-AtOEP34 at an N/P ratio (defined as the molar ratio of nitrogen in the cationic peptide to phosphate in the anionic pDNA) of 0.5 or 1 based on the compactness and surface charge of the complex (Supplementary Table S3). We then coated the complexes with the CPP BP100 to increase the translocation efficiency into the cell, as described in Thagun et al. (2019). To deliver the pDNA into plastids, we treated meristems collected from multiple shoots for each plant species with the peptide-DNA complex.

Using genomic DNA extracted from meristems several days after treatment, we first performed genotyping PCR at the plastid DNA-targeting locus to check the integration of the introduced pDNA into the plastid DNA via homologous recombination. For genotyping, we designed a pair of primers (L-Fw/Rv) to hybridize to the *aadA* marker gene and outside of the left homology arm to amplify the left-side junction region between the pDNA and plastid DNA (Figure 4A). Similarly, we designed another pair of primers (R-Fw/Rv) to hybridize to the *GFP* reporter gene and outside of the right homology arm to amplify the right-side junction region between the pDNA and plastid DNA (Figure 4A).

PCR genotyping of Arabidopsis successfully amplified a target 3.0-kb product (L-Fw/Rv) spanning the left junction region from pDNA-peptide-treated meristems, but not from untreated meristems (Figure 4B). Similarly, we amplified a target 2.7-kb product (R-Fw/Rv) spanning the right junction region only from pDNA-peptide-treated meristems (Figure 4B). We confirmed these genotyping PCR products from each junction region by sequencing. In addition, we previously confirmed no pDNA integration into plastid DNA without help of carrier peptides (Odahara et al. 2022; Yoshizumi et al. 2018), thus these results indicate that we successfully targeted plastid DNA with pDNA in treated Arabidopsis meristems. For *N. benthamiana* and *F.*×*ananassa*, genotyping analysis revealed efficient amplification of the target products spanning the left and right junction regions from pDNA-peptide-treated meristems but not from untreated meristems (Figure 4C, D). Nucleotide sequencing confirmed that these products are derived from each junction region, indicating that the pDNA was successfully integrated into plastid DNA of the treated meristems. Nevertheless, DNA gel blot analysis of *F.*×*ananassa* plastid DNA treated with peptide and pDNA showed no obvious band indicating integration of the pDNA into the target site (Supplementary Figure S3). This suggests the plastid DNA to be heteroplasmic for integration of the pDNA, and only a part of the plastid DNA likely to possess the pDNA. In summary, these results indicate that the pDNA was integrated into plastid DNA in the meristems of each plant species and was maintained heteroplasmically for at least several days.

### GFP production in plastids

**Figure 5:**
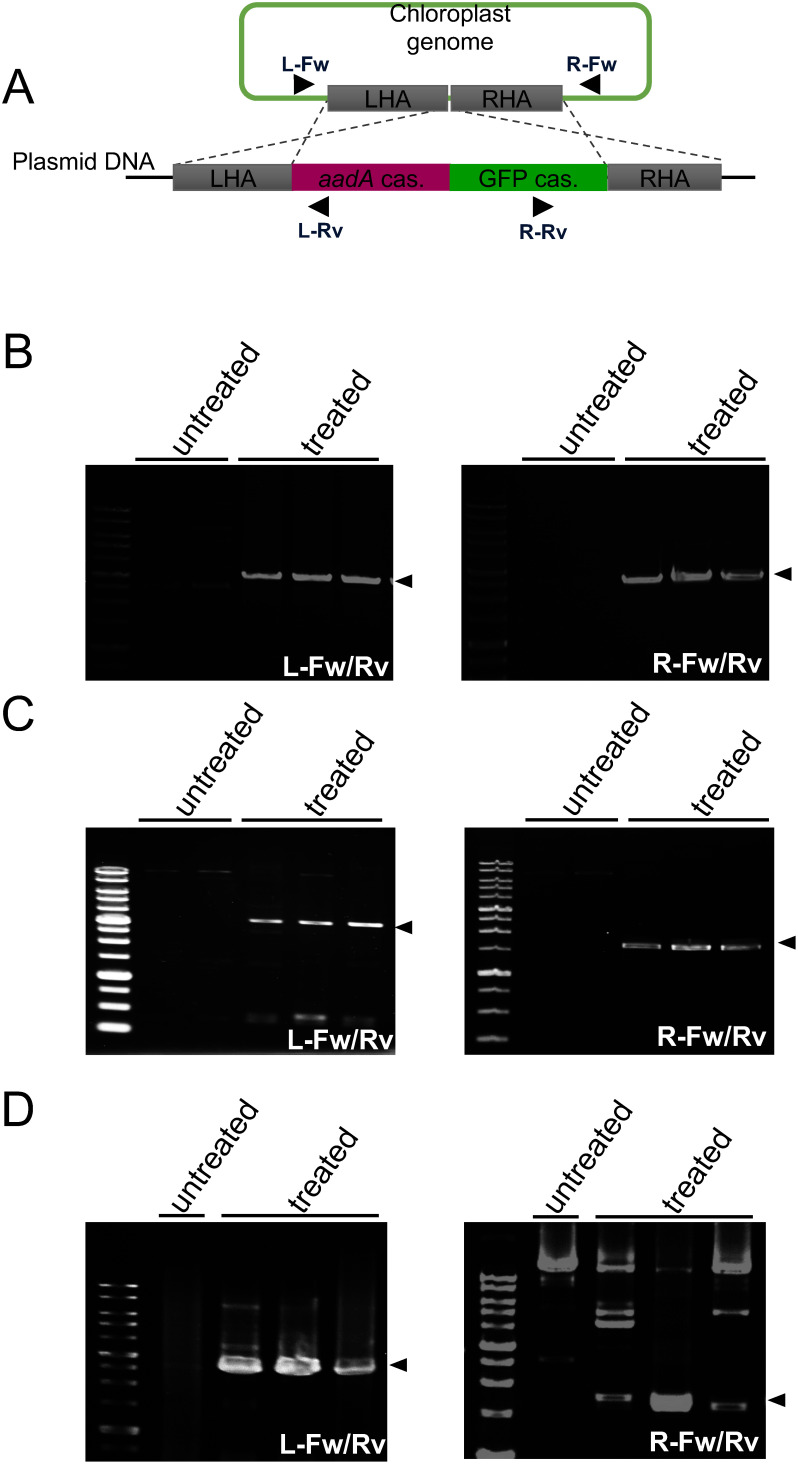
Figure 5. Imaging of GFP in *F.*×*ananassa* meristems. GFP localization in a meristem treated with DNA+peptide (upper panels) and the no DNA control meristem (lower panels). Chlorophyll autofluorescence (red) indicates plastids. Scale bars, 50 µm.

Since genotyping analysis showed the integration of the pDNA into plastid DNA in meristems, we examined gene expression from the integrated DNA construct. We first performed imaging of GFP signals in the plastids of meristems via CLSM. In *F.*×*ananassa* meristems treated with pDNA and peptide, 7±1 GFP signals appeared as punctate structures in the cells, and the GFP fluorescence co-localized with plastids in leaf primordia or outside of the shoot apical meristems (SAM; Figure 5). It was difficult to investigate the co-localization between GFP and plastids in the SAM due to lack of chlorophyll autofluorescence in this tissue (Yadav et al. 2019) and because P*_psbA_*, the promoter derived from *N. tabacum* that drove *GFP* expression, is expected to be inactive in such non-photosynthetic tissues. By contrast, we observed no GFP fluorescence in wild-type meristems (Figure 5). These results indicate that GFP accumulates in plastids of DNA/peptide-treated *F.*×*ananassa* meristems. As for *N. benthamiana*, we could not observe GFP fluorescence even in the meristems treated with pDNA and peptide.

**Figure 6:**
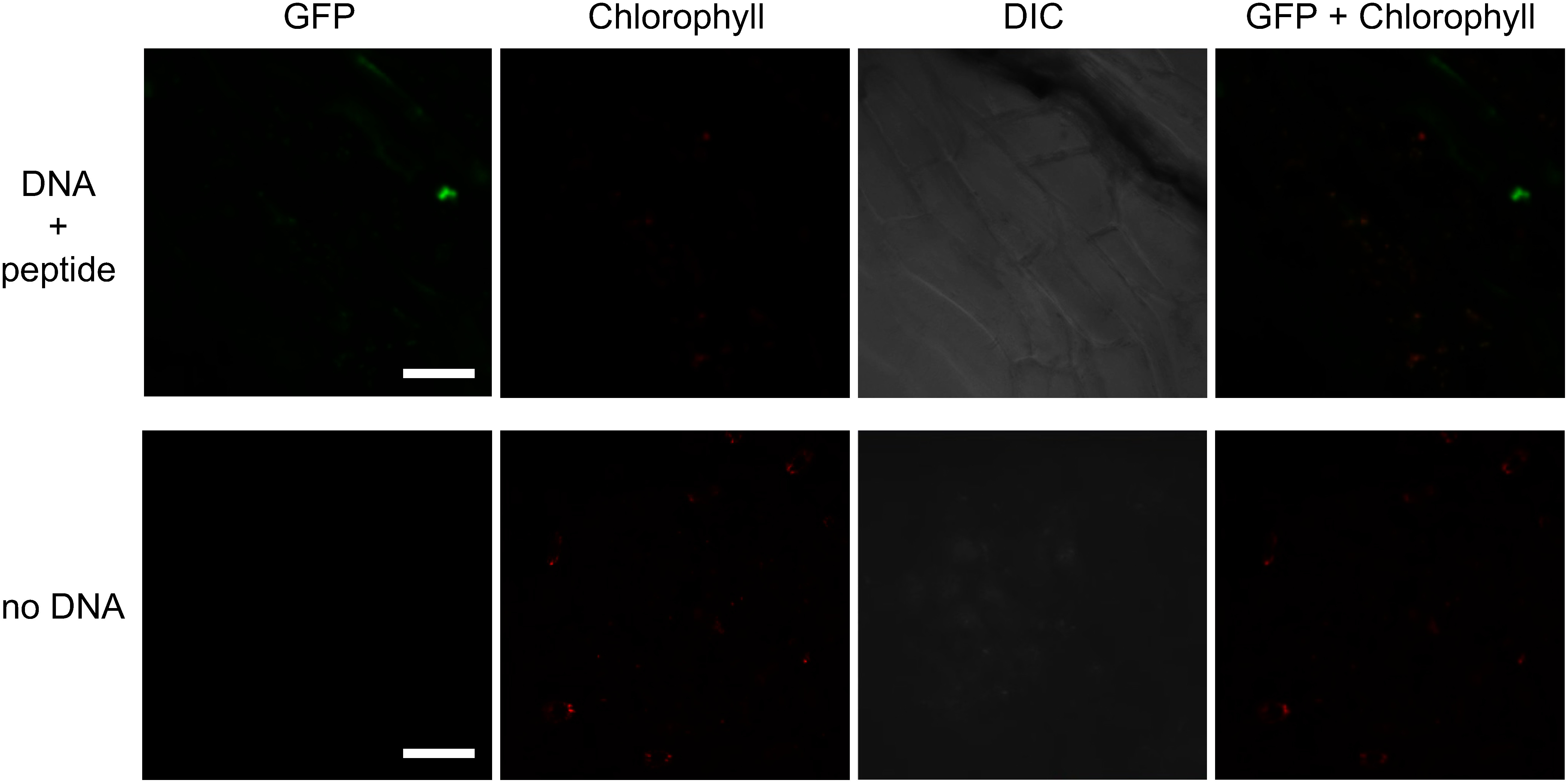
Figure 6. Immunoblot analysis of GFP in meristems. Immunoblot analysis for the presence of GFP using total proteins extracts from DNA-peptide-treated or untreated *N. benthamiana* and *F.*×*ananassa* meristems. GFP indicates the position of purified GFP (used as a marker).

We then analyzed GFP production in the meristems of *N. benthamiana* and *F.*×*ananassa* by immunoblotting using an anti-GFP antibody after affinity purification of GFP protein. We detected GFP accumulation in the meristems treated with the pDNA-peptide complex in *N. benthamiana* (Figure 6). However, although we detected GFP signals by CLSM, we barely detected any GFP in *F.*×*ananassa* meristems by immunoblot analysis (Figure 6). Perhaps pDNA delivery into *F.*×*ananassa* plastids was nonhomogeneous due to its large meristems. On the contrary, immunoblot analysis succeeded but CLSM failed detection of GFP in the *N. benthamiana* meristems, possibly due to homogeneous but low effciency pDNA delivery into *N. benthamiana* plastids, and the low GFP fluorescence should be indistinguishable from chlorophyll background. We failed detection of GFP in Arabidopsis meristems both by CLSM and immunoblot analysis, which might be due to low delivery efficiency caused by unsuitable CPP (Numata et al. 2018). Collectively, these results suggest that exogenous genes can be successfully expressed from pDNA in the plastids of peptide-treated meristems.

## Conclusions

Here, we established a liquid culture system for inducing multiple shoots in Arabidopsis, *N. benthamiana*, and *F.*×*ananassa*, and this offers a novel liquid culture method for induction of multiple shoots in these plant species, to our knowledge. Since the shoots were induced in liquid culture, we reasoned that they would be amenable to liquid-based gene delivery. Indeed, the multiple shoots induced using our system were suitable for our peptide-mediated gene delivery method, resulting in the targeted integration of the DNA construct into plastid DNA and expression of the reporter gene from the construct. This system, in combination with an appropriate selection system (e.g., spectinomycin or streptomycin resistance conferred by the *aadA* marker gene included in the DNA construct used in this study), should be suitable for in planta plastid transformation in the future. On the other hand, throughput of plastid transformation using leaf explants should be superior to the system shown here with regard to their throughput. Importantly, meristems are often used as targets for in planta nuclear transformation by clustered regularly interspaced short palindromic repeat (CRISPR)/CRISPR-associated nuclease 9 (Cas9)-mediated genome editing, since they do not require a tissue culture step and contain cells destinated to become germ cells (Imai et al. 2020). The efficient system for multiple shoot induction in liquid medium developed in this study should be suitable for high-throughput in planta nuclear transformation using genome editing.

## Abbreviations

BAP: 6-benzylaminopurine
CPP: cell penetrating peptide
GA_3_: gibberellic acid
KIN: kinetin
pDNA: plasmid DNA

## Description of Supplementary Files

This manuscript contains Supplemental Tables 1–3 and Figures 1–3.
