# Supplementary material for: A multiple shoot induction system for peptide-mediated gene delivery into plastids in Arabidopsis thaliana, Nicotiana benthamiana, and Fragaria×ananassa
Source: Plant Biotechnol (Tokyo). 2023 Dec 25;40(4):263–71. doi: 10.5511/plantbiotechnology.23.0501a (PMC10905367; doi:10.5511/plantbiotechnology.23.0501a)
Supplement: Supplementary Data [file plantbiotechnology-40-4-23.0501a-s001.pdf]

Supplementary Information

A multiple shoot induction system for peptide-mediated gene delivery into plastids in *Arabidopsis thaliana*, *Nicotiana benthamiana,* and *Fragaria* × *ananassa*

Masaki Odahara, Ara Most Tanziman, Remi Nakagawa, Shogo Ishio, Shinjiro Ogita, and Keiji Numata

Supplementary Table S1-3

Supplementary Figure S1-3

Supplementary Table S1. Amino acid sequences, molar mass, computed isoelectric point (pI) and net charge at pH 7.0 of the cationic domain-fused CTP KH-AtOEP34, and the CPP BP100. Underlined regions represent the chloroplast-targeting domain in the CTP and the cell-penetrating domain in the CPP.

Peptide	Amino acid sequence	Molar mass (g/mol)	Isoelectric point (pI)	Net charge at pH 7.0
KH-AtOEP34	KHKHKHKHKHKHKHK HKHMFAFQYLLVM	3650.4	11.2	9.9
BP100	KKLFKKILKYL	1421.8	10.9	5.0

Supplementary Table S2. List of primers used for genotyping PCR.

Plant species	Primer	Primer sequence	Product length (bp)
A. thaliana	L-Fw	CCGCTATGCCAAGCCAAAAG	3084
	L-Rv	CGTTGTCCCGCATTTGGTAC	
	R-Fw	ACATGAGCGTGAAAGGGGTT	2714
	R-Rv	TCTGCCCTTTCGAAAGATCCC	
N. benthamiana	L-Fw	CTCCCATTTCGAGTCAAGAAAAAAACGG	3300
	L-Rv	CTGGCGATGAGCGAAATGTAGTGC	
	R-Fw	CCCGAAGAGTAACTAGGACCAATTTAGTC	1700
	R-Rv	TCTGCCCTTTCGAAAGATCCC	
F. ananassa	L-Fw	GAAGACTGACACCCCCAAGT	3254
	L-Rv	TTCTCCGCGCTGTAGAAGTC	
	R-Fw	TCCAGAGGTTAAGCGTACTCT	1686
	R-Rv	TCTGCCCTTTCGAAAGATCCC	

Supplementary Table S3. Size (Z-Average) and zeta potential (ZP) analysis for various N/P ratio of plasmid-CTP/-CPP complexes.

pDNA	Complex	N/P	Z- Average (d.nm)	ZP (mv)	PDI
pPTTF_Fa	pDNA-CTP	0.5	136.1	-33.2	0.332
		1.0	180.7	-17.2	0.596
		1.5	237.0	-19.9	0.561
	pDNA-CTP-CPP	0.5	2283	4.70	0.403
		1.0	2415	-3.71	0.353
		1.5	1775	6.11	0.292
pPTTF_Nb	pDNA-CTP	0.5	103.6	-32.7	0.334
		1.0	151.0	-19.0	0.371
		1.5	260.6	39.5	0.641
pPTTF_At	pDNA-CTP	0.5	151.0	-19.0	0.371
		1.0	140.0	31.2	0.356
		1.5	260.6	39.5	0.641

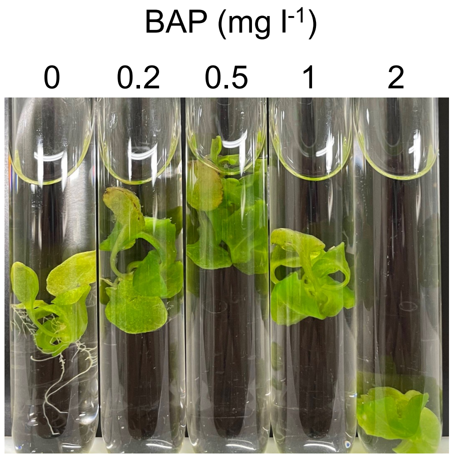

Supplementary Figure S1. Cultivation of Arabidopsis shoots in a medium containing maltose. Arabidopsis shoots were cultivated in Gamborg's B5 medium containing 3% maltose with various concentration of BAP.

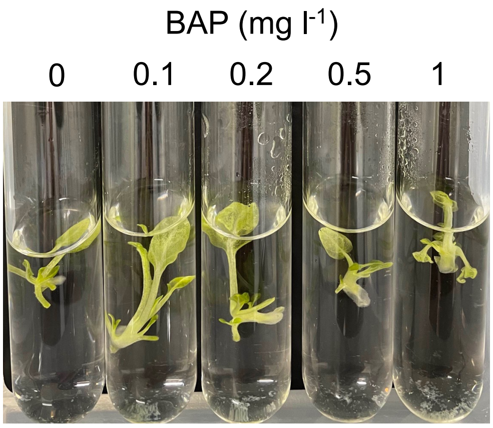

Supplementary Figure S2. Induction of *N. benthamiana* multiple shoots.
N. benthamiana shoots were cultivated in Gamborg's B5 medium containing 3% sucrose with various concentration of BAP.

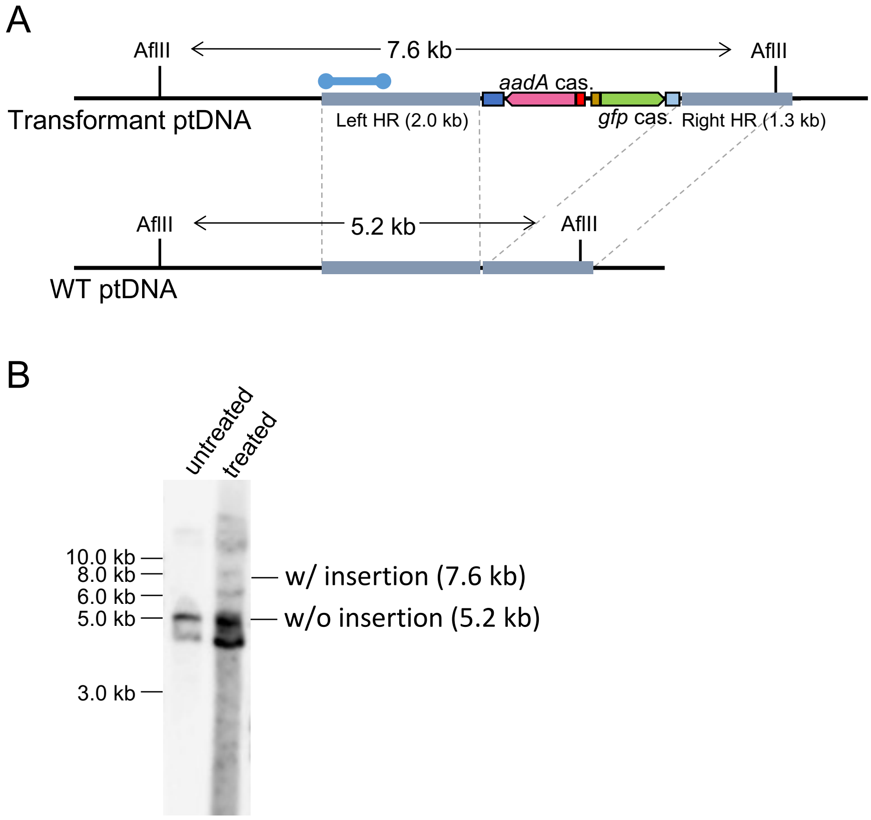

Supplementary Figure S3. DNA gel blot analysis of *F. × ananassa* plastid DNA in multiple shoot.
A. Maps of WT and transformant plastid DNA. Left and right homologous regions (HR) are shown by gray bold lines. A bold blue line represents the probe used in DNA gel blotting.
B. DNA gel blot analysis of plastid DNA. AflII-digested genomic DNA of *F. × ananassa* meristems treated with peptide and DNA construct was hybridized with the probe shown in (A).
